# Causal Association Between the Mucosal and Luminal Microbiotas from the Gastrointestinal Tract of Weaned Piglets Using Bayesian Network

**DOI:** 10.3390/microorganisms13020256

**Published:** 2025-01-24

**Authors:** Shu Yoshimura, Takamitsu Tsukahara, Toru Takahashi, Hiroto Miura, So Morishima, Masaaki Kise, Jiye Shin, Yoshihiro Yahara, Ryo Inoue

**Affiliations:** 1 Marubeni Nisshin Feed Co., Ltd., Tochigi 329-2763, Japan; yoshimura-s@mn-feed.com (S.Y.); kise-m@mn-feed.com (M.K.); shin-j@mn-feed.com (J.S.); yahara-y@mn-feed.com (Y.Y.); 2 Kyoto Institute of Nutrition & Pathology, Kyoto 610-0231, Japan; t-takahashi@nichiyaku.ac.jp; 3 Laboratory of Animal Science, Department of Applied Biological Sciences, Faculty of Agriculture, Setsunan University, Hirakata 573-0101, Japan; hiroto.miura@setsunan.ac.jp (H.M.); so_mrsm@yahoo.co.jp (S.M.); ryo.inoue@setsunan.ac.jp (R.I.)

**Keywords:** gastrointestinal microbiota, digesta, mucosa, Bayesian network, weaning pig

## Abstract

The aim of this study was to investigate the microbiota composition and its potential interactions across seven gut locations (stomachs, jejuna, ilea, ceca, proximal colons, distal colons, and recta) in weaned pigs to identify key influencing microbiotas. To compare between microbiota compositions, 16S rRNA gene amplicon sequencing was performed. Six 70-day-old healthy crossbred (Duroc × Large White × Landrace) piglets were introduced as donors. A Bayesian network (BN) was used to examine the directional interactions among the microbiotas evaluated (seven mucosal and seven digesta microbiotas). Based on edge connectivity frequency, the microbiota in jejunal mucosa was the central hub node, influencing other microbiotas, especially the mucosal microbiotas of the ileum, cecum, distal colon, and rectum. The jejunal mucosa was dominated by Prevotella and lactobacilli, both recognized for their contributions to pig health. Among Prevotella, Prevotella copri and Prevotella sp. were predominant in jejunal mucosa (4.6% and 2.9%, respectively). Lactobacilli, including eight distinct species, were distributed throughout the gastrointestinal tract. Notably, Ligilactobacillus salivarius and Lactobacillus amylovorus, known as immune-enhancing bacteria, were abundant in jejunal mucosa (1.0% and 0.8%) and digestas (0.9% and 19.2%), respectively. The BN identified rectal mucosa and digestas as two terminal nodes, influenced by upstream microbiotas in the gastrointestinal tract. This finding supports the link between fecal microbiota and pig productivity, as the fecal microbiota, closely resembling the rectal microbiota, reflects the conditions of the microbiota throughout the gastrointestinal tract.

## 1. Introduction

The intestinal microbiota plays a fundamental role in the host’s homeostasis and health [1]. With respect to pigs, the statuses of their intestinal microbiotas are crucial because they affect several physiological parameters, such as intestinal structure, intestinal barrier function, and immune system development [2]. It is worth noting that the intestinal microbiota is reportedly associated with pig productivity parameters, such as growth [3,4,5,6], fatness [7], and reproductive performance [8,9,10]. The composition of the microbiota varies across the alimentary tract because it is influenced by environmental factors such as nutrient availability, pH, redox potential and peristaltic frequency [11].

The mucosa has mucin layers that separate the epithelium from the digesta-associated microbiota (DAM) and harbor mucosa-associated microbiota (MAM) themselves [12]. It is known that the microbiota within the mucosa is unique, hence differing from that in the digesta [13]. Previous studies have suggested a relationship between the MAM and the host’s gut health [14] and immunity [15]. For example, Lu et al. [15] reported that the gene expression of immune cells, such as Toll-like receptor 4 in the blood and the concentrations of secretory immunoglobulin A in the colonic mucosae, correlated with the statuses of the colonic MAM of pigs.

Although the effect that the microbiotas in the mucosa and digesta, including that in feces, exerts on pig productivity is now recognized [16], there are still only a handful of studies reporting comprehensive comparisons between the microbiotas of the porcine mucosa and the digesta found throughout their gastrointestinal tracts [13,17]. For example, De Rodas et al. [17] analyzed the mucosal and luminal microbiotas of pigs from farrowing to finishing, but their results were limited, as they only analyzed the ilea, ceca, and colons, but excluded the stomachs, jejuna, and recta. Therefore, to the best of our knowledge, to date, no studies have conducted a comprehensive comparison of the microbiotas in the mucosa and the digesta from the stomachal-to-rectal area of pigs.

In previous studies investigating microbiota throughout the porcine gastrointestinal tract, microbiotas at each location were treated as independent ecosystems. However, as they harbor a contiguous space, there is no doubt that there are mutualistic relations between the microbiotas. Therefore, in the present study, causal relations among the microbiotas were evaluated using a Bayesian network (BN). The BN is categorized as a supervised machine learning algorithm [18], developed for industrial fields since the 1990s. The BN can detect causal relations between variables using Bayes’ theorem with stochastic predictions, eliminating researchers’ arbitrariness [18]. The results of BN are often represented by a graphical model. The graphical model can show the smooth visualization of causal relations among microbiotas using arrows and nodes. The comprehensive relationships of microbiotas can be simplified and visualized on a graphical model. In the present study, a graphical model was also employed to show the results of the BN.

To assess the relationships between them, we compared the microbiota compositions between the mucosa and the digesta in seven intestinal locations, namely the stomachs, jejuna, ilea, ceca, proximal colons, distal colons, and recta of weaned pigs. Next, a BN was conducted to examine the directional interactions among the microbiotas evaluated (seven mucosal and seven digesta microbiotas) to identify which microbiota could potentially influence other microbiotas.

## 2. Materials and Methods

### 2.1. Animals and Feeding Conditions

The experimental animals were handled as per the guidelines for animal studies of the Experimental Animal Committee of the Kyoto Institute of Nutrition & Pathology, Inc. (Kyoto, Japan; Approval No. 19033NP). In the present study, to avoid the maternal effect on the microbiotas, six healthy crossbred (Duroc × Large White × Landrace) piglets were chosen from four different litters raised in the Marubeni Nisshin Feed experimental farm (Tochigi, Japan). After weaning (21–22 days of age; mean body weight 6.4 ± 0.16 kg), all piglets were placed in a single pen (5.5 m^2^) in an open room. Piglets were fed two commercial feeds formulated by Marubeni Nisshin Feed (Tokyo, Japan). First, a pre-starter feed (Premium-Multi Prestarter, Marubeni Nisshin Feed, Tokyo, Japan) was given for 14 days after weaning. Afterwards, a starter feed (High-Skill Starter, Marubeni Nisshin Feed, Tokyo, Japan) was given for 36 days. The chemical compositions of the respective feeds are shown in Supplementary Table S1. All pigs had ad libitum access to feed and water during this study. All piglets (mean body weight 31.0 ± 0.77 kg) were euthanized for the collection of digestas and mucosae on day 50 after weaning.

### 2.2. Dissection Procedure

Anesthetics, namely 0.4 mg/kg of body weight (BW) of midazolam (Teva, 5 mg/mL; Takeda Pharma, Aichi, Japan), 0.07 mg/kg of BW of medetomidine hydrochloride (Dorbene, 1 mg/mL; Kyoritsu Seiyaku Corp., Tokyo, Japan), and 0.2 mg/kg of BW of butorphanol (Vetorphale, 5 mg/mL; Meiji Seika Pharma, Tokyo, Japan) were injected intramuscularly. Around 10 min post-injection, additional amounts of (1) midazolam (0.25 mg/kg of BW) and (2) medetomidine hydrochloride (0.05 mg/kg of BW) and butorphanol (0.2 mg/kg of BW), were intravenously and intramuscularly injected, respectively. While under deep anesthesia, the bodies of piglets were midline incised, and blood was collected from the cecal, ileal and portal veins, as well as from the abdominal aorta and the abdominal vena cava. The blood samples were used for a separate study. Next, the whole gastrointestinal tracts were dislodged from the carcasses, and the stomachs, the small intestines, the ceca, the proximal colons (*Gyri centripetales*), the distal colons (*Gyri centrifugales*), and the recta were individually separated with string ligations. The small intestines (SIs) were then sub-segmented into eight parts, as previously described [19]. The second SI sub-segment was considered as the jejuna and the eighth sub-segment as the ilea [19]. Digestas were collected from the stomachs, the jejuna, the ilea, the ceca, the proximal colons, the distal colons, and the recta. Digesta samples were then immediately cooled on ice. The mid-portion of each gastrointestinal part was washed with sterile phosphate-buffered saline (PBS) and mucosal samples were collected by scraping them with sterilized glass slides. The mucosal samples were immediately immersed in RNA*later* (ThermoFisher, Waltham, MA, USA) and cooled on ice. The cooled samples were then frozen and kept at −20 °C, transported to a laboratory at the Kyoto Institute of Nutrition & Pathology (Kyoto, Japan) within 24 h post-dissection and stored at −80 °C until further use.

### 2.3. Analysis of the Mucosal and Digesta Microbiotas by 16S rRNA Gene Amplicon Sequencing

Bacterial DNA was extracted from the thawed samples (digestas and mucosae) using a previously reported procedure [20]. Briefly, bacterial DNA was extracted by a commercial extraction kit (QuickGene DNA tissue kit; KURABO, Osaka, Japan) as previously described. Twenty-five milligrams of homogenized digesta and mucosae samples were collected into sterilized 2 mL screw-cap tubes containing zirconia beads; 250 µL of a tissue lysis buffer, which was part of the kit, was then applied to the homogenized digesta, and the suspensions were smashed twice at 3000 r.p.m. for 2 min by a Micro Smash MS-100 apparatus (Tommy, Tokyo, Japan). Twenty-five microliters of proteinase K, being part of the kit, was added to the suspensions and incubated at 55 °C for 2 h. After centrifugation (15,000× *g*, 10 min, 25 °C), 200 µL of the supernatant were transferred to new microtubes. One hundred and eighty microliters of the lysis buffer, being part of the kit, was applied to the supernatants. After incubation at 70 °C for 10 min, 240 µL of ethanol (99% *v*/*v*; SIGMA-Aldrich Japan, Tokyo, Japan) were added. The bacterial genomic DNA in the suspension was purified by using a QuickGene 810 system (KURABO).

Library preparation and 16S rRNA gene amplicon sequencing by a MiSeq apparatus (Illumina, San Diego, CA, USA) were carried out as described by Inoue et al. [21]. Briefly, the V3–4 region of 16S rRNA genes in each sample was amplified by primers 341F and 805R containing a 5′ overhang adapter sequence for PCR by a KAPA HiFi HotStart Ready Mix (Kapa Biosciences, Wilmington, MA, USA). The amplicon was purified with NucleoFast 96 PCR plates (Takara Bio Inc., Shiga, Japan). A second PCR was carried out using the KAPA HiFi HotStart Ready Mix to attach a unique combination of dual indices (I5 and I7 indices) and Illumina sequencing adapters to each sample. The amplicon of the second PCR was purified and the concentration was normalized using a SequalPrep Normalization Plate Kit (Life Technologies, Tokyo, Japan). Each of the normalized amplicons was then evenly pooled and concentrated using AMPure XP beads (Beckman Coulter, Tokyo, Japan). The quantity of the library was assessed with a Library Quantification Kit for Illumina (Kapa Biosciences). The library was denatured with 0.2N NaOH (SIGMA-Aldrich Japan) and combined with phiX Control (v3, Illumina, San Diego, CA, USA; expected 20%). Eleven picometers of the library, combined with phiX Control, was heat-denatured at 96 °C for 2 min and sequenced using a 285 bp paired-end strategy on the Miseq apparatus (Illumina) as per the manufacturer’s instructions.

After the sequencing, quality filtering, denoising, determination of amplicon sequence variants (ASVs), and taxonomic classification of ASVs against the SILVA 138 database, the obtained reads were analyzed by QIIME2 (ver. 2020.8) with DADA2 plugin. Denoising using DADA2 was conducted with the trimming length from the left set at 17 and that from the right at 19. The truncation length was set at 250 for both reads. Singletons and ASVs assigned to chloroplasts and mitochondria were removed in this study. The creation of a phylogenetic tree was conducted using SATé-enabled phylogenetic placement [22]. The sequence of ASVs belonging to the genus *Prevotella* and lactobacilli were further analyzed with BLASTn to identify the nearest known species to each ASV.

### 2.4. Statistical Analysis

The α-diversity Chao1 and Shannon indices were calculated with QIIME2. Depending on the results of the Bartlett test, the α-diversity indices and the relative abundances (%) of bacterial genera between the pig groups (segments × mucosa or digesta) were statistically compared with a one-way ANOVA or the Kruskal–Wallis test. Tukey or Steel-Dwass post hoc comparisons were carried out as needed. Differences were considered significant if *p* < 0.05. The values are expressed as the means ± the standard errors. The β diversity was calculated based on the weighted UniFrac distance metrics. It should be noted that the genus “*Lactobacillus*” in the microbiome analysis was adopted before the reclassification in 2020 [23], whereas “*Lactobacillus*” in BLASTn research analysis was adopted after reclassification.

The causal effects were calculated using a Bayesian network [18]. A Bayesian network can show causations between variables using arrows on a graphical model. Lines and arrowheads in a figure indicate causes and effects, respectively. The Bayesian network was analyzed using R software version 4.2.3 (The R Project for Statistical Computing, Vienna, Austria) and Rstudio version 2023.09.0+463.

## 3. Results

### 3.1. Diversity in Gut Microbiota Across Within-Segment and Between-Segment Comparisons

The α- and β-diversities of gut microbiotas throughout the gastrointestinal tract are shown in Supplementary Figure S1.

Unlike the Shannon index, the Chao1 index differed both within segments (between mucosa and digesta in same location) and between segments (between different locations). The Chao1 indices in digestas in the jejuna, ceca and recta were the highest, followed by those in mucosae in the ceca and recta, and digestas in stomachs. By contrast, the Chao1 indices in digestas in the ilea, and proximal and distal colons were low.

From the PCoA plots based on β-diversity, MAM and DAM were clearly divided. The DAMs were subdivided into two clusters: DAM in the upper alimentary tract (stomachs, jejuna, and ilea) and in the lower alimentary tract (ceca, colons, and recta). However, between segments, the MAMs were not clearly subdivided.

### 3.2. Directional Relations Among Microbiotas

**Figure 1:**
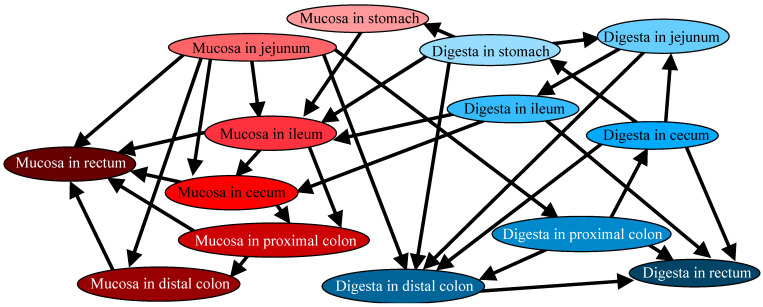
Causal relations of variables analyzed by a Bayesian network between the inter- and intra-regions of the alimentary tracts of weaned pigs. Lines and arrowheads in a figure indicate causes and effects, respectively. All datasets were ordinal values. Red and blue nodes indicate mucosae and digestas, respectively, and their color gradient from light to dark indicates upper to lower segments.

The directional relations among microbiotas are shown in Figure 1. The results of the BN showed that microbiotas in the proximal segments, such as the jejuna, were located upstream of distal segments, such as colons. Indeed, the microbiotas of jejunal mucosae were the most connected upstream hub node; directly connected with the MAM in most segments (ilea, ceca, proximal and distal colons, and recta) and the DAM in proximal and distal colons. The direct connection within segments was observed only in the stomachs and ilea; in both segments, DAM was the cause of MAM. In other segments, no apparent within-segment interactions were found, suggesting that the microbiotas throughout the gastrointestinal tract were under complex interactions. Both the MAM and DAM in the rectum were the two end nodes and hence, not located upstream of any nodes.

### 3.3. Genera Abundance in Gut Microbiota Within and Between Segments

At the genus level, the abundances of 49 bacterial genera with a percentage higher than 1% within and between segments were significantly different (Supplementary Table S2). Although the 49 genera were significantly different within and between segments of the alimentary tracts, as per the Kruskal–Wallis test, no significant differences were observed between the segments, as per the post hoc comparison analysis.

The abundances of all phyla and genera are shown in Supplementary Tables S3 and S4. Firmicutes (now known as Bacillota) were the predominant phylum in all segments, regardless of whether it was a MAM or DAM.

By abundance, the top three genera in the stomach and ileal MAM were *Prevotella*, *Lactobacillus*, and *Helicobacter*. In the jejunal MAM, the three most abundant genera were *Prevotella*, an unknown genus from *Lachnospiraceae*, and *Anaerovibrio*. In the cecal and proximal colon MAM, the top three most abundant genera were *Prevotella*, *Campylobacter*, and *Helicobacter*. The top three genera in the MAM in the distal colon were *Prevotella*, *Campylobacter*, and *Chlamydia*, and this pattern extended to the MAM in the rectum. In the stomach DAM, the top three most abundant genera were *Lactobacillus*, *Actinobacillus*, and *Streptococcus*. In the jejunal DAM, the top three genera were *Lactobacillus*, *Streptococcus*, and an unknown order from class Bacilli. For the ileal DAM, the top three genera were *Lactobacillus*, *Streptococcus*, and *Clostridium_sensu_stricto_1*, with this pattern continuing in the rectal DAM. In the cecal DAM, the top three most abundant genera were *Lactobacillus*, *Streptococcus*, and *Subdoligranulum*, which extended to the DAM in the proximal colon. The top three genera in the distal colon DAM were an unknown genus from the family *Muribaculaceae*, *Lactobacillus*, and *Streptococcus*.

### 3.4. Genus Prevotella and Lactobacilli

**Figure 2:**
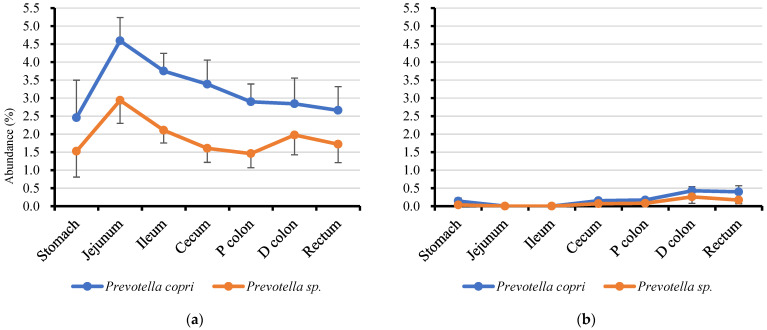
Dominant *Prevotella* distribution in the inter- and intra-regions of the alimentary tracts of weaned pigs. (**a**) Mucosa-associated; (**b**) digesta-associated. The symbols represent the means, and the bars represent the standard errors (*n* = 6).

**Figure 3:**
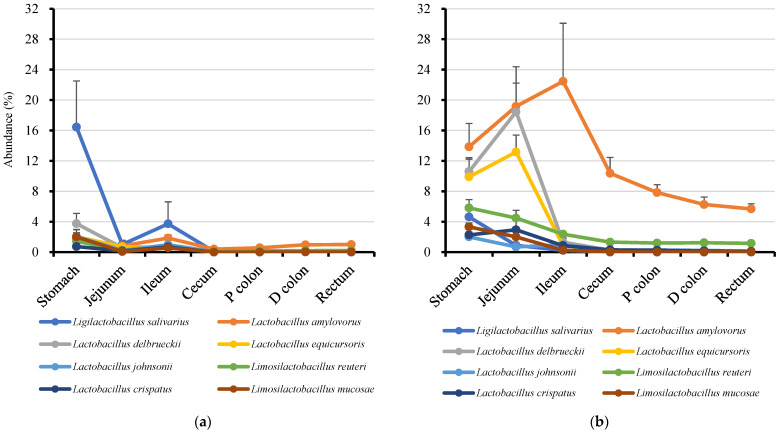
Dominant lactobacilli distribution in the inter- and intra- regions of the alimentary tract of weaned pigs. (**a**) Mucosa-associated; (**b**) digesta-associated. The symbols represent the means, and the bars represent the standard errors (*n* = 6).

Genus *Prevotella* and lactobacilli, the most abundant bacteria in jejunal mucosae and all alimentary tracts, respectively (Supplementary Table S2), are likely part of the core bacteria in the porcine gut microbiota. Therefore, as expected, species profiles of *Prevotella* and lactobacilli were observed within and between segments, as shown in Figure 2 and Figure 3.

When we extracted the most dominant (top 10 abundant) *Prevotella* ASVs in the jejunal mucosae, all dominant *Prevotella* were detected in all segments of the mucosae, and the distal regions of digestas (ceca, proximal and distal colons and recta). By contrast, the most dominant lactobacilli ASVs in the jejunal mucosae were of the following types: (1) mainly detected in the mucosae, (2) detected equally in the mucosae and digestas, (3) mainly detected in the digestas. The nearest known lactobacilli species inhabiting the mucosae was *Ligilactobacillus salivarius*, whereas the lactobacilli in the digesta were *Lactobacillus amylovorus*, *Lactobacillus delbrueckii*, *Lactobacillus equicursoris*, *Limosilactobacillus reuteri*, *Lactobacillus crispatus*, and *Limosilactobacillus mucosae*. The nearest other known lactobacilli species equally detected in the mucosae and digestas was *Lactobacillus johnsonii*.

## 4. Discussion

The porcine MAM and DAM in seven locations in the gastrointestinal tract were analyzed and their directional interactions were evaluated by a BN. As in previous work [12,13], the MAMs and DAMs were clearly different even within segments in all of the locations evaluated. With regard to the differences between segments, the differences were clearer in DAMs than in MAMs; both the α and β diversities of DAMs between the upper and the lower gastrointestinal tracts were different.

The BN revealed that the MAM in the jejuna could influence both the MAMs and DAMs in many segments, but especially the MAMs. This is partly supported by a report by Duarte and Kim [24] that showed a stronger correlation of the MAM in the jejunum, than with the fecal microbiota, with serum protein carbonyl (a marker of oxidative stress) and tumor necrosis factor-α concentrations, suggesting that the jejunal MAM links with porcine physiological conditions were stronger than that of the latter with fecal microbiota. We theorized that the MAM in the jejuna could be a key microbiota in the porcine gastrointestinal tract, at least in weaned pigs. The suggestion regarding the importance of the jejunal MAM carried an implication regarding the effect of antimicrobial treatment on the gut microbiota. When injected intramuscularly, some antimicrobials are secreted to the intestine by gut efflux and/or bile [25]. As bile is secreted from the duodenum, the jejunal MAM can be significantly affected and this effect may spread to other MAMs and DAMs through directional interactions between microbiotas. This theory can explain the fact that even one intramuscular injection of antimicrobials can significantly affect the “fecal” microbiota [26].

In contrast to jejunal MAM, rectal MAM and DAM were identified as two downstream-located microbiotas, suggesting that these microbiotas are influenced by many other microbiotas in the gastrointestinal tract proximal to the rectum. In this context, fecal microbiota is thought to reflect at least the conditions of DAMs throughout the gastrointestinal tract, as it is known that rectal DAM closely resembles the fecal microbiota. This concept strengthens the rationale for using feces to investigate the relationship between gut microbiotas and pig productivity. It has been a controversial topic whether fecal microbiota is optimal for evaluating the overall gut microbiota condition, as it does not closely resemble the microbiotas in the proximal segments of the gastrointestinal tract, as shown in the present work and other studies [16]. However, considering its ease of collection, non-invasive nature, and the above-mentioned findings, feces can be regarded as a useful tool for such investigations. With respect to the rectal MAM, it also seems useful to predict the conditions of MAMs throughout the gastrointestinal tract. However, as it is unclear whether the rectal mucosa can be collected stably and non-invasively and without slaughter, this point needs further elucidation.

*Prevotella*, the highest detected genus in jejunal mucosa, is known as a key member of the intestinal microbiota in pigs [27,28]. *Prevotella* is also known as a mucus-associated bacterium that is able to degrade mucin [27,29] and tolerate oxygen [30]. From our results, 10 ASVs were detected as major *Prevotella* in the jejunal mucosa; most of all ASVs (7/10) were identified as the *Segatella copri* (previously known as *Prevotella copri*) by the BLASTn database. *Segatella copri* is known to be highly abundant in the porcine gut after weaning [27]. It is also associated with growth performance at the distinct production stages of pigs (suckling, weaning, growing, and finishing) [31]. *Prevotella* colonization could also be detected in the rectal mucosa and partially detected in the rectal digestas.

Lactobacilli was detected to be dominant in all regions of MAMs and DAMs. Lactobacilli is well known in the swine industry for their beneficial effects. Therefore, probiotic strains of lactobacilli are marketed worldwide [32]. Unlike *Prevotella*, many lactobacilli species were detected in MAMs and DAMs, but they were more abundant in DAMs than in MAMs. *Ligilactobacillus salivarius*, and *Lactobacillus amylovorus*, which were the most abundant lactobacilli in the jejunal MAM and DAM, respectively, are known as an immune-enhancing bacterium in the porcine mucosa [33], and a beneficial bacterium for porcine development [34,35], respectively. *L. amylovorus* is considered to be a potential probiotic for pigs. Moreover, in the present work, although it was found that some *L. amylovorus* species could adhere to the porcine epithelial cells [36], it was detected more in the digesta than in the mucosa.

## 5. Conclusions

The BN suggested that the microbiotas in jejunal mucosae were the key microbiotas, influencing many other MAMs and DAMs in the porcine gastrointestinal tract. The genus *Prevotella* and lactobacilli seem to be part of the core bacteria and important in the porcine alimentary tracts; these bacteria are well known to contribute to the health of pigs. While two species of *Prevotella*, such as *P. copri* and *Prevotella* sp., were detected mainly in the DAMs, eight species of lactobacilli were detected in the DAMs and MAMs, all throughout the gastrointestinal tract. Particularly, *L. salivarius* and *L. amylovorus*, known as immune-enhancing bacteria in the porcine mucosa, were the most abundant lactobacilli in the jejunal MAM and DAM. We theorized that, at least in weaned pigs, the microbiota in the rectal digesta, and perhaps also in the mucosae, could be a useful sample for investigating the relation between the gut microbiota and the host’s physiology. This is because they reflect the conditions of other MAMs and DAMs proximal to the rectum and can be collected relatively easily, non-intensively and without slaughter. Further investigation is needed to corroborate the interaction of the within-segment and between-segment microbiotas in the porcine gastrointestinal tract.

## Data Availability

The sequence data have been deposited in the NCBI Sequence Read Archive (SRA) under accession number PRJNA755590. The datasets generated and/or analyzed during the current study are available from the corresponding author upon reasonable request.

## Supplementary Materials

The following supporting information can be downloaded at: https://www.mdpi.com/article/10.3390/microorganisms13020256/s1, Table S1: Estimated chemical composition of diets; Table S2: Genera abundances that significantly differed in the gut microbiotas between the inter- and intra-regions of the alimentary tracts of weaned pigs. Table S3: Phylum abundances in the gut microbiotas within and between segments of the alimentary tracts of weaned pigs. Table S4: Genus abundances in the gut microbiotas within and between segments of the alimentary tracts of weaned pigs. Figure S1: α and β diversities of gut microbiotas in porcine mucosa and digestas.
