# Supplementary material for: Causal Association Between the Mucosal and Luminal Microbiotas from the Gastrointestinal Tract of Weaned Piglets Using Bayesian Network
Source: Microorganisms. 2025 Jan 24;13(2):256. doi: 10.3390/microorganisms13020256 (PMC11858346; doi:10.3390/microorganisms13020256)
Supplement: Supplementary file 1 [file microorganisms-13-00256-s001.zip › Yoshimura_pig microbiota Suppl Figures.pptx]

## Slide 1
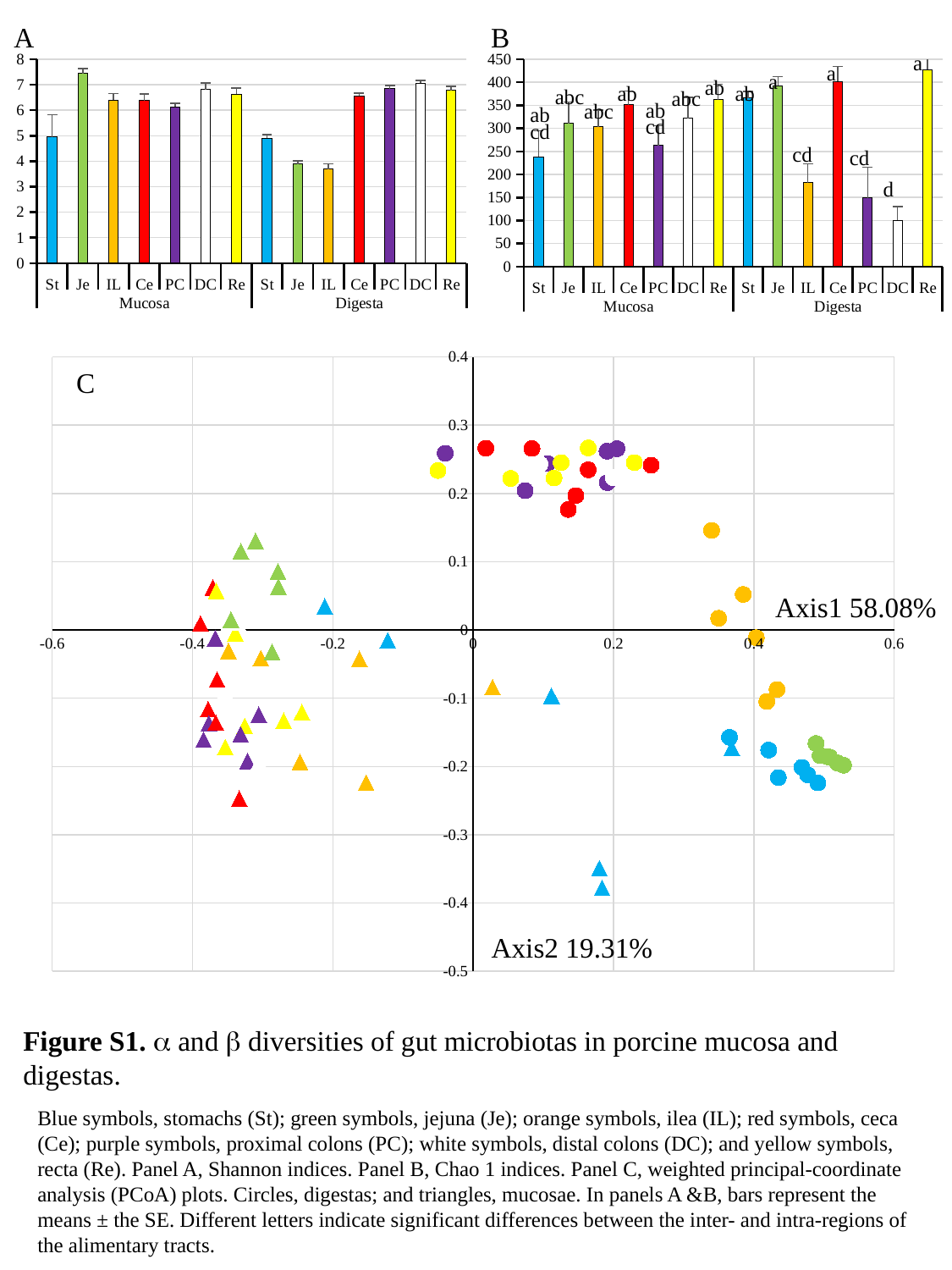

B
A
a
### Chart
| Category | Shannon |
|---|---|
| St | 4.951651571204463 |
| Je | 7.443955407585309 |
| IL | 6.400977775642145 |
| Ce | 6.383486549179632 |
| PC | 6.125056946029272 |
| DC | 6.808330600760603 |
| Re | 6.62083943735655 |
| St | 4.900003497417018 |
| Je | 3.8963661341805 |
| IL | 3.6851154518287235 |
| Ce | 6.5599636616679495 |
| PC | 6.846858597806311 |
| DC | 7.057605747791442 |
| Re | 6.771804824859971 |
### Chart
| Category | chao1 |
|---|---|
| St | 238.37555555555537 |
| Je | 311.17936507936497 |
| IL | 303.82619047619016 |
| Ce | 351.2476551226548 |
| PC | 263.22499999999997 |
| DC | 322.05448343079917 |
| Re | 362.34990379990364 |
| St | 365.44444444444434 |
| Je | 392.2416666666666 |
| IL | 182.16666666666666 |
| Ce | 401.7555555555555 |
| PC | 149.33333333333334 |
| DC | 99.91666666666667 |
| Re | 427.2083333333333 |a
a
ab
ab
ab
abc
abc
abc
ab
cd
ab
cd
cd
cd
d
### Chart
| Category | PC2 |
|---|---|C
Axis1 58.08%
Axis2 19.31%
Figure S1. a and b diversities of gut microbiotas in porcine mucosa and digestas.
Blue symbols, stomachs (St); green symbols, jejuna (Je); orange symbols, ilea (IL); red symbols, ceca (Ce); purple symbols, proximal colons (PC); white symbols, distal colons (DC); and yellow symbols, recta (Re). Panel A, Shannon indices. Panel B, Chao 1 indices. Panel C, weighted principal-coordinate analysis (PCoA) plots. Circles, digestas; and triangles, mucosae. In panels A &B, bars represent the means ± the SE. Different letters indicate significant differences between the inter- and intra-regions of the alimentary tracts.
